# Femoral and pelvic osteotomies for severe hip displacement in nonambulatory children with cerebral palsy: a prospective population-based study of 31 patients with 7 years’ follow-up

**DOI:** 10.1080/17453674.2019.1675928

**Published:** 2019-11-01

**Authors:** Terje Terjesen

**Affiliations:** Department of Orthopaedic Surgery, Oslo University Hospital, Rikshospitalet, Oslo, Norway

## Abstract

Background and purpose — There is no consensus regarding the optimal treatment of hip displacement in children with cerebral palsy (CP). This prospective study assessed the outcome of femoral and pelvic osteotomies for severe hip displacement in nonambulatory children and analyzed prognostic factors for outcome.

Patients and methods — 31 nonambulatory children (20 boys), recruited from a population-based screening program, consecutively underwent unilateral (23) or bilateral (8) osteotomies and bilateral soft-tissue releases at a mean age of 6.1 years (2.2–9.9). The procedures were femoral varus osteotomy alone (20 hips) and combined Dega-type pelvic osteotomy and femoral osteotomy (19 hips). Final outcome was termed good if the patient had not undergone further bony surgery and migration percentage (MP) was < 50%. The mean follow-up time was 7.1 years (3.8–11).

Results — The mean preoperative MP was 69% (36–100). The outcome was good in 22 patients (29 hips) and poor in 9 patients (10 hips). Mean time to failure was 3.6 years (1.0–6.0). GMFCS level V and high MP 1-year postoperatively were statistically significant risk factors for poor final outcome. There was a higher rate of good outcome after combined osteotomies compared with isolated femoral osteotomy, but the difference was not statistically significant (p = 0.2).

Interpretation — Better primary correction was obtained after combined femoral and pelvic osteotomies than after isolated femoral osteotomy, indicating that combined osteotomies are the preferred method in hips with the most severe degrees of displacement. Prophylactic femoral osteotomy of the contralateral non-subluxated hip is hardly indicated.

There is no consensus as to the optimal surgical treatment of hip displacement in children with cerebral palsy (CP). Preventive surgery with soft-tissue releases usually provides a satisfactory outcome in ambulatory children with moderate degrees of displacement, whereas the outcome deteriorates in nonambulatory children with more pronounced displacement (Shore et al. 2012, Terjesen 2017). In this group more radical surgery such as femoral and pelvic osteotomies is indicated. Although good results have been published after these procedures (McNerney et al. 2000, Oh et al. 2007), several matters are still under discussion: optimal age at operation, whether femoral osteotomies alone or combined femoral and pelvic osteotomies should be performed, and whether contralateral prophylactic femoral osteotomy should be done when the contralateral hip is not displaced (Valencia 2010, Shore and Graham 2017).

The present study is a prospective population-based study of children enrolled in the Norwegian CP registry. Previously, data from this registry have been used to evaluate the ability of soft-tissue surgery to prevent deterioration of subluxation (Terjesen 2017). The present study evaluates the outcome of femoral and pelvic osteotomies in nonambulatory children with severe hip displacement. The aims of the study were to answer the following questions:

1. What is the outcome of reconstructive hip osteotomies combined with soft-tissue releases in nonambulatory children?
2. Is there any difference in outcome between hips with combined femoral and pelvic osteotomies and hips with isolated femoral osteotomy?
3. Are there any predictors for good and poor outcome?
4. Is prophylactic femoral osteotomy of the contralateral non-subluxated hip indicated?

## Patients and methods

Patients in this prospective study were recruited from the screening program for children with CP in south-east Norway, designated CPOP (Cerebral Parese Oppfølgings Program). 31 children (20 boys) born during 2002–2006 had consecutively undergone 39 femoral and/or pelvic osteotomies (8 bilaterally) during the period 2007–2014. The mean age at surgery was 6.1 years (2.2–9.9). 23 patients had not been operated on previously, whereas 8 patients had previously undergone bilateral soft-tissue release, but had relapse of their hip displacement. All the children were nonambulatory. The functional classification according to the Gross Motor Function Classification System (GMFCS) (Palisano et al. 1997) was level IV in 8 children and level V in 23. 29 children had bilateral spastic CP (quadriplegia in 23 children and diplegia in 6) and 2 children had dyskinesia (variable muscle tone). 11 patients had intrathecal baclofen therapy.

The hospital’s case records were insufficient for evaluation of hip pain because of missing information. Thus, the data on pain were prospectively registered, using the information from the yearly clinical examinations at the child habilitation centers. Physiotherapists filled out a standardized form, which included whether the patients or caregivers had noticed any pain during the last 4 weeks, and the location of pain was noted. Unfortunately, the side was not specified.

### Radiographic evaluation

An anteroposterior radiograph of the pelvis and hip joints was taken with the child in the supine position. Care was taken to position the child correctly with the legs parallel and to avoid rotation of the pelvis. The radiographic measurements were performed by the author, who has many years of experience in evaluating radiographs of children’s hips.

The following radiographic parameters were measured: migration percentage (Reimers 1980), acetabular index (Hilgenreiner 1925), and pelvic obliquity. Migration percentage (MP) is the percentage of the femoral head lateral to the acetabulum (lateral to Perkins’ line), measured parallel to Hilgenreiner’s line. The hips were classified as normal (MP < 33%), subluxation (MP 33–89%), and dislocation (MP ≥ 90%) (Reimers 1980). Acetabular index (AI) is the angle between the line through the medial and lateral edges of the acetabular roof and Hilgenreiner’s line. Pelvic obliquity was measured as the angle between the horizontal line and the line between the lowest points of the pelvic bones on the right and left side. Angles < 3° were not registered as pelvic obliquity.

According to the study protocol a pelvic radiograph should be taken once a year. Thus, the progression in MP per year pre- and postoperatively could be assessed. Reduction in MP caused by the operation (“primary” correction) was defined as difference between preoperative MP and MP 1-year postoperatively.

### Operative procedures

Preoperatively, 16 patients had unilateral hip displacement (MP > 33%) and they underwent unilateral bony surgery. Thus, prophylactic contralateral osteotomies were not performed. 15 patients had bilateral displacement, of whom 8 patients underwent bilateral bony surgery. In the remaining 7 patients, unilateral bony surgery of the worst hip was performed.

Choice of osteotomies (femoral osteotomy alone or combined femoral and pelvic osteotomies) varied according to the surgeons’ preferences, but usually combined osteotomies were chosen in complete dislocations and for the most severe subluxations. Proximal femoral varus osteotomy alone was performed in 13 patients (20 hips) and combined osteotomies were done in 18 patients (19 hips). Children with combined osteotomies had higher age at surgery, higher preoperative MP, better primary correction, more often intrathecal baclofen pump, and more often unilateral osteotomies (Table 1).

**Table 1.** Association between types of osteotomy and various clinical and preoperative radiographic variables

Variables	FVO **^a^**	Combined osteotomies **^b^**	p-value
Girls	7	4	0.07
Boys	6	14	
GMFCS level IV	4	4	0.6
GMFCS level V	9	14	
Primary operation	12	11	0.05
Secondary operation	1	7	
Unilateral operation	7	16	0.03
Bilateral operation	6	2	
Baclofen pump	2	9	0.05
No baclofen pump	11	9	
Hip pain preoperatively	5	5	0.5
No hip pain preoperatively	8	13	
Age at surgery (SD)	5.2 (1.7)	6.7 (1.7)	0.02
Migration percentage (SD)			
preoperative	63 (15)	75 (18)	0.02
1 year postoperatively	27 (14)	19 (12)	0.09
reduction	36 (24)	56 (21)	0.008
Acetabular index preoperative (SD)	30 (3.6)	30 (3.3)	0.8
Pelvic obliquity preoperative (SD)	3.1 (3.2)	4.6 (4.9)	0.3

a Femoral varus osteotomy.

b Femoral varus osteotomy and pelvic osteotomy.

GMFCS = gross motor function classification system.

SD = standard deviation.

**Figure 1.**
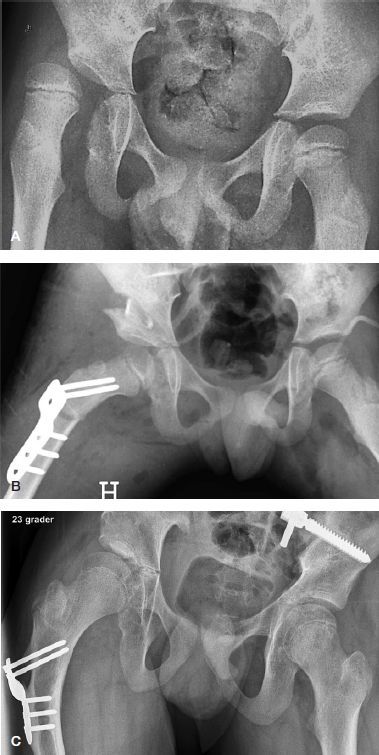
A. Preoperative radiograph of a boy, aged 7.0 years and GMFCS level V, with severe subluxation of his right hip (MP 67%). B. 1 day after femoral osteotomy (varus, derotation and shortening), pelvic osteotomy, and bilateral soft tissue releases, showing good femoral head coverage bilaterally. The cortical bone segment removed from femur has been used as autograft in the open wedge of the pelvic osteotomy. C. 7.4 years postoperatively, at an age of 14.4 years, showing satisfactory femoral head coverage bilaterally, MP 19% (right hip) and 0% (left hip)

The surgical procedures were done with the patient in the supine position, and an image intensifier was used. Femoral varus osteotomy was performed through a longitudinal lateral approach. A transverse femoral osteotomy just above the lesser trochanter was performed with an oscillating saw. The osteotomy was a combination of varization, derotation, and shortening. One Kirschner wire drilled transversely into the femur proximal to the osteotomy and one distal to the osteotomy were used as guide wires for derotation. A neck–shaft angle of 110–120° and derotation of about 30° were aimed at. In 24 osteotomies a second transverse osteotomy distal to the first was performed, and a wedge of bone was removed, aiming for a femoral shortening of 1–2 cm. The osteotomy was fixed with a 110° pediatric locked compression plate (LCP; Synthes, Switzerland) (Rutz and Brunner 2010) in 30 osteotomies (Figure 1) and a 90° AO blade plate in 2. In the remaining 7 osteotomies a straight plate with 2 screws in each fragment was used, after the plate had been bent according to the planned varization.

For the pelvic osteotomy, a modification of the incomplete transiliac Dega osteotomy was performed (Grudziak and Ward 2001) through a transverse anterior incision approximately 2 cm distal to the superior anterior iliac spine. The anterior part of the iliac apophysis was split and the inner and outer tables of the ilium were subperiosteally exposed. The osteotomy was performed with curved osteotomes. It started just above the anterior inferior iliac spine and proceeded posteriorly, keeping about 1.5 cm above the attachment of the joint capsule. The direction of the osteotomy was medially and inferiorly and ended just above the horizontal limb of the triradiate cartilage, leaving the posterior part of the cortex at the sciatic notch intact. A broad osteotome was used to lever open the osteotomy laterally and anteriorly. The bone graft from the femoral shortening was inserted in the open wedge (Figure 1).

In the 23 patients without previous soft-tissue releases, simultaneous bilateral tenotomies of adductor longus, gracilis, and iliopsoas were performed, using a short oblique incision over the origin of adductor longus. Postoperatively, a plaster cast with moderate hip abduction was used for 6 weeks in 22 children and an abduction orthosis was used in 9 children.

### Assessment of outcome

The outcome at the last follow-up was graded into 2 categories according to a modification of the classification of Shore et al. (2015), with both the patient (n = 31) and the hip (n = 39) as the unit of analysis. The outcome was termed good (“success”) when MP at the last follow-up was < 50%. If final MP was ≥ 50% and/or the patient had undergone subsequent bony surgery (pelvic and/or femoral osteotomies) to improve femoral head coverage, the outcome was poor (“failure”). In addition, the clinical outcome was assessed by the information on pain at the last CPOP follow-up. For those who underwent reoperation, pain evaluation at the last examination before reoperation was used.

### Statistics

SPSS (version 25) was used for the statistical analysis (IBM, Armonk, NY, USA). Categorical variables were analyzed with the Pearson chi-square test. Continuous variables were analyzed with Student’s t-test. Potential risk factors for poor final outcome were estimated as relative risks using univariable Poisson loglinear regression and multivariable exact logistic regression. All tests were 2-sided. Differences were considered significant when the p-value was < 0.05. The percentage survival according to postoperative time (years) was described with a Kaplan–Meier plot.

### Ethics, funding, and potential conflicts of interest

The study was approved by the Regional Committee of Medical Research Ethics (no. 2012/2258) and the hospital’s Privacy and Data Protection Officer. No external funding was received for this study and there are no conflicts of interest.

## Results

**Figure 2.**
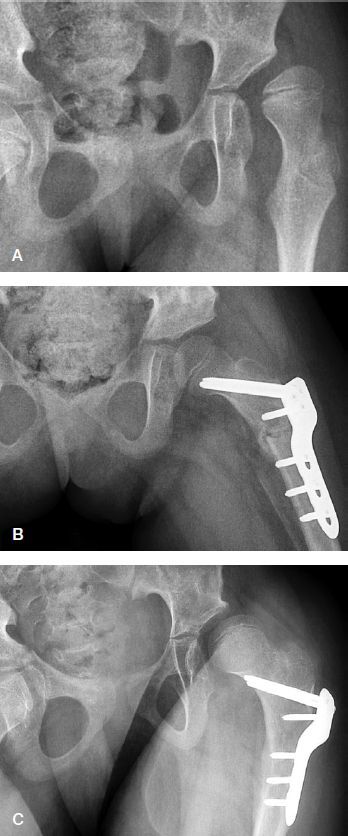
A. Preoperative radiograph of a girl, aged 8.1 years and GMFCS level V, with severe subluxation of her left hip (MP 74%). B. 6 weeks after femoral and pelvic osteotomies of the left hip and bilateral soft tissue releases, showing good femoral head coverage. C. 2.9 years postoperatively (age 11.0 years), showing relapse of subluxation of her left hip (MP 51%).

The mean follow-up time of children who have not undergone subsequent hip surgery was 7.1 years (3.8–11) and their mean age at the latest follow-up was 13.6 years (11.3–16.6). When the outcome of the worst hip was used in patients with bilateral bony procedures, the outcome was good in 22 patients and poor in 9 patients (Figure 2). When hip was used as the unit of analysis, a good outcome was achieved in 29 out of 39 hips.

Potential risk factors for poor final outcome were analyzed with univariable Poisson loglinear regression (Table 2). When the variables with p-value < 0.2 were analyzed with multivariable exact logistic regression, GMFCS level V and high MP 1 year postoperatively were independent risk factors for poor outcome (Table 3). The rate of good outcome after combined osteotomies was higher (good results in 15 of 18 patients) than that after femoral osteotomy alone (good results in 7 of 13 patients), but the difference was not statistically significant (p = 0.2). 2 patients died 3.7 and 6.2 years, respectively, after the index operation.

**Table 2.** Potential prognostic factors for poor radiographic outcome in 31 patients, estimated as relative risks (RR) for failure, using univariable Poisson loglinear regression

Variables	Outcome		Statistical analysis RR (95% CI)	p-value
	Good	Failure		
Girls	7	4	Reference	
Boys	15	5	0.7 (0.2–2.6)	0.6
GMFCS, level IV	8	0	Reference	
GMFCS, level V^a^	14	9	OR 6.3 (0.8–∞)	0.08
Primary operation	15	8	Reference	
Secondary operation	7	1	0.4 (0.1–2.9)	0.3
Unilateral operation	18	5	Reference	
Bilateral operation	4	4	2.3 (0.6–8.6)	0.2
No pain preoperatively	15	6	Reference	
Pain preoperatively	7	3	1.1 (0.3–4.2)	0.9
Combined osteotomies	15	3	Reference	
Femoral osteotomies	7	6	2.8 (0.7–11.1)	0.2
Age (SD)	6.4 (1.9)	5.4 (1.8)	0.8 (0.6–1.2)	0.3
Migration percentage (SD)				
preoperative	72 (19)	69 (12)	1.0 (0.96–1.03)	0.8
1 year postoperatively	21 (11)	35 (14)	1.05 (1.01–-1.1)	0.03
Acetabular index preop. (SD)	30 (3.0)	31 (3.8)	1.08 (0.88–1.3)	0.5
Pelvic obliquity preop. (SD)	3.4 (3.7)	5.9 (5.8)	1.1 (0.94–1.3)	0.3

For abbreviations, see Table 1.

a Exact logistic regression was used because one group contained the value “0.”

**Table 3.** Multivariable analysis of odds ratios for poor outcome, including variables with a p-value < 0.2 in univariable analysis, using multivariable exact logistic regression

Variables	Odds ratio	95% CI	p-value
GMFCS, level V	15	1.4–∞	0.02
Femoral osteotomy	5.4	0.5–237	0.2
Migration percentage 1 year postop.	1.1	1.0–1.4	0.01

For abbreviations, see Table 1. CI = confidence interval.

The mean preoperative MP was 69% (36–100), 72% in hips with unilateral osteotomies and 65% in those with bilateral procedures. Only 4 of the operated hips had mild or moderate subluxation (MP 36–48%). The remaining 35 hips had severe subluxation (MP ≥ 50%) or complete dislocation. MP during the entire postoperative period was larger in hips with poor final outcome (Table 4). The progression in MP (mean change per year) was pronounced (15%) during the mean preoperative observation period of 2.9 years, with no statistically significant difference between hips with good and poor outcome. The mean postoperative progression was less than 1% per year in hips with good outcome and 10% per year in hips with poor final outcome (Table 4).

**Table 4.** Migration percentage pre- and postoperatively (mean values with 95% confidence intervals (CI) in parentheses) of all 39 hips and comparison between hips with good and poor radiographic outcome

Migration percentage	All hips	Outcome		Difference		p-value **^a^**
		Good	Poor	Mean	(95% CI)	
Preoperative	69 (63–75)	69 (62–76)	68 (60–77)	0.8	(–12 to 14)	0.9
Primary correction^b^	46 (38–54)	49 (39–59)	36 (22–49)	13	(–4 to 31)	0.1
1 year postop	23 (19–28)	20 (16–24)	33 (22–43)	– 13	(–22 to –4)	0.008
3 years postop	30 (24–36)	25 (21–30)	47 (27–67)	–22	(–34 to –10)	0.04
5 years postop	33 (24–43)	25 (18–33)	58 (32–83)	–32	(–49 to –15)	0.001
Progression per year						
preoperative^c^	15 (11–19)	14 (10–19)	16 (6–25)	–1.0	(–11 to 9)	0.8
postoperative	2.9 (1–5)	0.7 (–0.4–1.8)	10 (5–15)	–9.5	(–15 to –4)	0.002
Contralateral side						
preoperative	29 (24–34)					
last follow-up	26 (16–35)					

a Student’s t-test.

b Difference between migration percentage preoperative and 1 year postoperatively.

c Progression of preoperatively was calculated during a mean period of 2.9 years (0.8–5.8).

**Figure 3.**
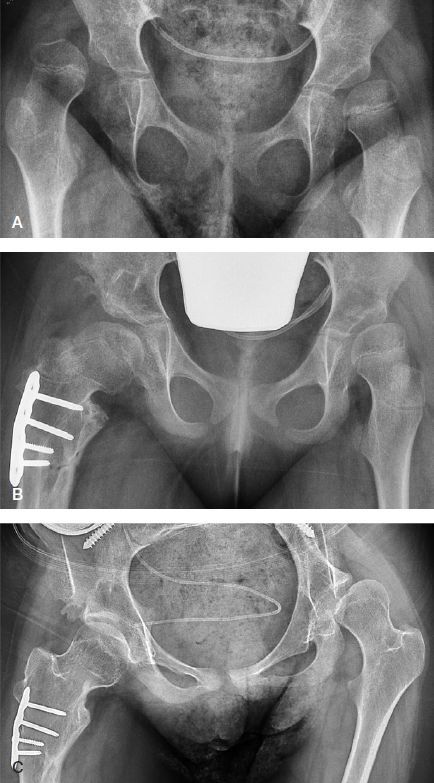
A. Preoperative radiograph of a girl, aged 5.7 years and GMFCS level V, with complete dislocation of her right hip (MP 100%) and subluxation of her left hip (MP 51%). B. 14 months after femoral and pelvic osteotomies of the right hip and bilateral soft tissue releases, showing slight subluxation of both hips (MP right hip 36% and left hip 37%). C. 7.3 years postoperatively (age 13.0 years), showing good position of right hip and deterioration of left hip (MP 64%).

In the 23 patients with unilateral osteotomies, the contralateral hip deteriorated in 3 patients. The surgical guidelines had not been followed in 1 of these patients, because contralateral soft-tissue releases had not been performed. 2 patients had subluxation with preoperative MP 48% and 51%, respectively, on the contralateral side, and these hips deteriorated postoperatively (Figure 3). Of the remaining 5 patients with contralateral subluxation (MP 36–50%) but no contralateral bony surgery, postoperative normalization occurred in 4 patients (MP < 33%) whereas subluxation remained unchanged (MP 38% preoperatively and 40% at follow-up) in 1 patient. None of the contralateral hips with preoperative MP < 33% and bilateral soft-tissue releases developed subluxation during follow-up.

Preoperative hip pain had been noted in 10 of the 31 children. 1 year postoperatively these patients were painless, but 6 of the other patients had hip pain. At the last follow-up, pain was significantly more frequent in patients with poor radiographic outcome (5 of 9 patients) than in patients with good outcome (2 of 22 patients; p = 0.005).

Postoperative complications occurred in 4 patients. 2 children had failure of the femoral fixation and re-dislocation of the osteotomy 1–3 months postoperatively, which was treated with plate re-fixation in 1 child and plaster cast in the other. Decubitus ulcer of the heel because of pressure from the plaster and pneumonia occurred in 1 patient each. In addition to these early complications, femoral fracture of the operated extremity occurred 1–5 years postoperatively in 4 patients. The fractures were treated with plate fixation. There were no cases of avascular necrosis of the femoral head.

**Figure 4.**
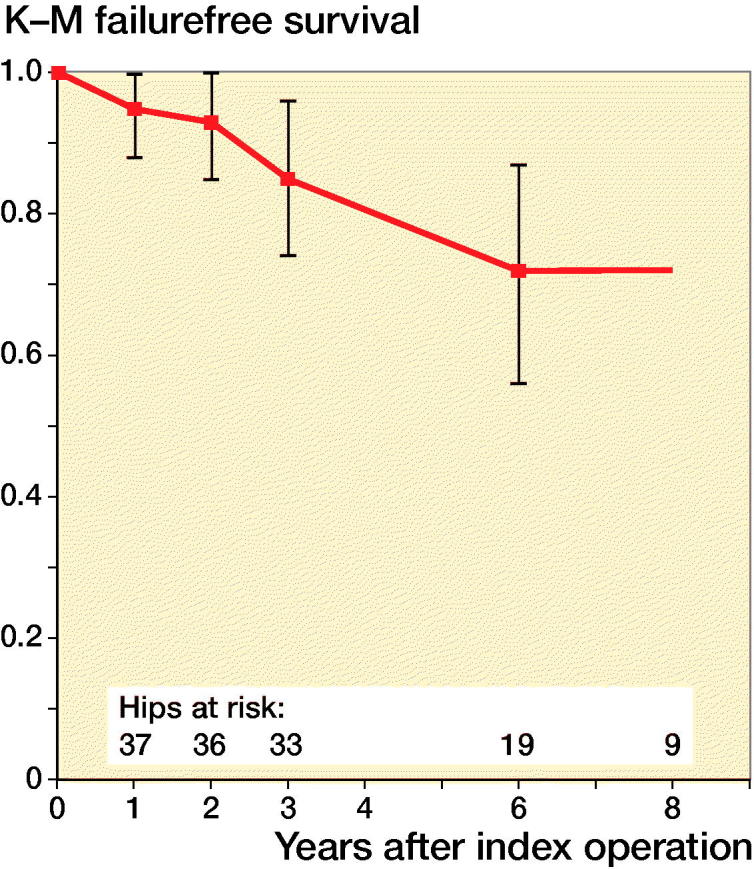
Kaplan–Meier survival plot (% survival with 95% confidence intervals) in all 39 hips, with time to failure (reoperation or MP ≥ 50%) as “survival”.

The mean time to failure was 3.6 years (1.0–6.0). There was a shorter mean time to failure after femoral varus osteotomy (3.1 years) than after combined osteotomies (5.0 years), but the difference was not statistically significant (p = 0.2). Kaplan–Meier survival analysis with time to failure as endpoint showed that survival fell from 95% 1 year postoperatively to 74% at 6 years (Figure 4). No obvious reason for failure could be identified in 5 patients (6 hips). The reason was poor primary correction in 3 patients. 1 child had repeated femoral fractures of the operated side and also had respiratory problems; therefore, proximal femoral resection was performed after his third fracture. Reoperation has been performed in 5 hips (1 femoral osteotomy, 2 pelvic osteotomies, 1 combined pelvic and femoral osteotomies, and 1 proximal femoral resection). The mean period from index operation to reoperation was 3.6 years (2.3–6.3). 5 hips have so far not undergone further surgery; their mean MP at the last follow-up was 69% (51–100).

## Discussion

Reconstructive osteotomies are indicated in children with severe hip displacement, but there is no consensus as to which surgical strategy is preferable. Since the outcome deteriorates with increasing length of follow-up and with decreasing functional capacity (Shore et al. 2015), nonambulatory children with a follow-up of > 5 years should be separately analyzed, as was done in Table 5 (see Supplementary data). The median failure rates were 31% (22–25) after isolated femoral osteotomy and 15% (9–20) after combined femoral and pelvic osteotomies. Previous reports have recommended combined osteotomies in patients with severe subluxation or dislocation and pronounced dysplasia of the acetabulum (Brunner and Baumann 1997, McNerney et al. 2000, Sankar et al. 2006, Oh et al. 2007). This is in accordance with the strategy of the present study, where hips with combined osteotomies had larger preoperative displacement and better primary correction than those with femoral osteotomy alone. Pelvic osteotomy is, however, an extensive procedure and represents a large surgical trauma to a severely involved patient with respiratory or other medical problems. Therefore, the indications for pelvic osteotomy were based on the surgeon’s preference after discussion of benefits and risks with the parents and child neurologists.

Shore et al. (2015) found better outcome at GMFCS levels I–III than in nonambulatory children at levels IV/V. Different results have been published regarding GMFCS level IV versus level V. Whereas failure rates at 5 years of 15% at GMFCS level IV and 24% at level V were reported by Shore et al. (2015), failure rates of 26% in both GMFCS levels IV and V were found by Zhang et al. (2014). In the present study, level V was a significant risk factor for poor outcome. The fact that no failures occurred after femoral osteotomies in children at GMFCS level IV indicated that femoral osteotomy alone should be considered in patients with this grade of function; however, the number of patients is too small to draw definite conclusions. In GMFCS level V, the outcome after femoral osteotomy was poor in 7 of 14 hips, indicating that combined osteotomies would be the preferred procedure.

High preoperative MP was a risk factor for poor outcome in some studies (Oh et al. 2007, Settecerri and Karol 2000, Rutz et al. 2015) but neither in the present study nor in the multivariate analysis by Shore et al. (2015). High MP 1 year postoperatively, which was a significant risk factor in the present study, seems not to have been assessed in previous studies.

Age was not a prognostic factor in the present study and in 2 previous studies (Settecerri and Karol 2000, Oh et al. 2007), whereas young age (< 6 years) was a risk factor of failure in other studies (Brunner and Baumann 1997, Ruzbarsky et al. 2013, Shore et al. 2015). When the patients are followed in a population-based screening program, there is no reason to postpone surgery when the MP exceeds 40–50%, no matter the age of the patient. It was therefore not unexpected that the age of the present patients was in the lower range compared with other studies (Table 5).

The need for concomitant open reduction is controversial. Some authors recommended open reduction (Jozwiak et al. 2000, McNerney et al. 2000, Sankar et al. 2006) whereas others did not perform open reduction (Settecerri and Karol 2000, Mallet et al. 2014). Capsulotomy and open reduction were not used in the present study because clinical experience and peroperative fluoroscopy indicated that the hips were sufficiently contained by osteotomies and soft-tissue releases.

Opinion differs with regard to surgical strategy in patients with a normal contralateral hip. Whereas some studies recommended prophylactic, concurrent femoral osteotomy of the contralateral hip to obtain pelvic balance (Barakat et al. 2007, Oh et al. 2007) others recommended unilateral osteotomy only (Settecerri and Karol 2000, Larsson et al. 2012). After unilateral soft-tissue or bony surgery in 27 nonambulatory patients with no subluxation of the contralateral side, deterioration of the contralateral side occurred in 19 hips, leading the authors to caution against unilateral surgery (Noonan et al. 2000). When unilateral osteotomy was combined with bilateral soft-tissue releases, the rate of contralateral deterioration was only 8% (Larsson et al. 2012). This is in keeping with the present results. A decision analysis (Park et al. 2012) estimated that the contralateral hip should be prophylactically operated if the rate of later instability was ≥ 27%. Shukla et al. (2013) found that lack of contralateral soft-tissue release and preoperative MP > 25% were risk factors for contralateral subluxation. In the present study, no contralateral deterioration was seen when the preoperative MP was less than 33% and the surgical guidelines had been followed. Therefore, a reasonable conclusion would be that contralateral prophylactic femoral osteotomy is not indicated if bilateral soft-tissue releases are performed.

There is a trend to deterioration of outcome with increasing follow-up time. Shore et al. (2015) reported failure rates of 24% at 5 years and 42% at 10 years after osteotomies in children at GMFCS level V. The mean time to failure in the present study was 3.6 years at GMFCS level V and there were no failures later than 6 years postoperatively. Zhang et al. (2014) reported a somewhat longer mean time to failure (5 years). If the hip remains stable during the first postoperative years, there is a relatively small risk of later deterioration. The mean postoperative progression in MP was only 0.7% per year in hips with good outcome in the present study, which is similar to the progression rate of 1.1% after combined osteotomies (Mallet et al. 2014).

Pain relates strongly to quality of life and is therefore an important outcome parameter. A recent population-based study in children with CP found, at a mean age of 9.5 years, hip pain in 14% of children with normal hips and in about 60% of children with severe subluxation (Ramstad and Terjesen 2016). The present results showed that operative treatment had a good effect on hip pain, in agreement with previous studies (Barakat et al. 2007, Rutz et al. 2015).

The reported rate of postoperative complications after hip reconstructions varies considerably, from 0% to 81% (Ruzbarsky et al. 2013). In a systematic review (Hesketh et al. 2016), the AVN rate varied from 0% to 46%. These rates are difficult to compare because different classifications were used and because of mixed populations of patients. The complication rate was moderate in the present study. There were no cases of avascular necrosis of the femoral head, which is in accordance with some other studies (Sankar et al. 2006, Mallet et al. 2014). Not performing capsulotomy and open reduction might have contributed to the low rate of avascular necrosis.

There are some limitations of the study. First the number of patients was small, which could reduce the validity of the statistical analysis. The choice of osteotomies was not randomized, but based on the surgeon’s preference. Pain was reported crudely and without a validated scoring system. Pain assessment in severely involved CP patients is difficult because the children have limited ability to express the presence and intensity of pain. Information from caregivers is necessary, although such information could also be somewhat unreliable. The strengths of the study are that it is population-based and prospective, with a follow-up of all the children.

## Conclusions

1. The outcome was good in 22 of 31 patients (29 of 39 hips) after reconstructive hip osteotomies in nonambulatory children with severe hip displacement.
2. There was a higher rate of good outcome after combined osteotomies compared with isolated femoral osteotomy, but the difference was not statistically significant (p = 0.2).
3. Significant risk factors for a poor final outcome were markedly decreased functional level (GMFCS level V) and high migration percentage 1 year postoperatively.
4. Routine prophylactic femoral osteotomy of the contralateral non-subluxated hip is hardly indicated.

## Supplementary data

Table 5 is available as supplementary data in the online version of this article, http://dx.doi.org/10.1080/17453674.2019.1675928

## Supplementary Material

Supplemental Material
