# Supplementary material for: Femoral and pelvic osteotomies for severe hip displacement in nonambulatory children with cerebral palsy: a prospective population-based study of 31 patients with 7 years’ follow-up
Source: Acta Orthop. 2019 Nov 1;90(6):614–21. doi: 10.1080/17453674.2019.1675928 (PMC6844395; doi:10.1080/17453674.2019.1675928)
Supplement: Supplemental Material [file IORT_A_1675928_SM6593.pdf]

Supplementary data

Table 5. Medium to long-term outcome (≥ 5 years) of femoral vs. combined femoral and pelvic osteotomies in nonambulatory children with CP

Author (year)	No. of hips	GMFCS levels	Age	Follow-up time (years)	Migration percentage pre-operative	1 year post-operatively	final follow-up	Failures (%) [a]
Femoral osteotomies								
Noonan et al. (2001)	70	IV/V	7.6	5.2	82	19	28	28
Larsson et al. (2012)	23	IV/V	7.6	5.0	65		34	22
Zhang et al. (2014)	39	IV/V	5.0	5.2	48	0		33
Present study (2019)	20	IV/V	5.2	7.0	63	26	36	35
Median of 4 studies			6.4	6.1	64	19	34	31 (22–35)
Combined femoral and pelvic osteotomies								
Sankar et al. (2006)	11	IV/V	10.6	16.7				9
Oh et al. (2007)	25	IV/V	8.9	10.8				20
Mallet et al. (2014)	20	IV/V	8.1	9.1	55	15	5	15
Zhang et al. (2014	19	IV/V	5.0	5.2	69	0		11
Present study (2019)	19	IV/V	6.7	6.9	76	18	28	16
Median of 5 studies			8.1	9.1	69	15	17	15 (9–20)

GMFCS = gross motor function classification system.
[a] Re-subluxation and/or reoperation.
